# Do all sedatives promote biological sleep electroencephalogram patterns? A machine learning framework to identify biological sleep promoting sedatives using electroencephalogram

**DOI:** 10.1371/journal.pone.0304413

**Published:** 2024-07-02

**Authors:** Sowmya M. Ramaswamy, Merel H. Kuizenga, Maud A. S. Weerink, Hugo E. M. Vereecke, Sunil B. Nagaraj, Michel M. R. F. Struys

**Affiliations:** 1 Department of Anesthesiology, University Medical Center Groningen, University of Groningen, Groningen, The Netherlands; 2 Department of Anesthesiology and Reanimation, AZ St.-Jan Brugge Oostende AV, Brugge, Belgium; 3 School of Physics, Maths and Computing, Computer Science and Software Engineering, The University of Western Australia, Perth, Australia; 4 Department of Basic and Applied Medical Sciences, Ghent University, Gent, Belgium; Belgrade University Faculty of Medicine, SERBIA

## Abstract

**Background:**

Sedatives are commonly used to promote sleep in intensive care unit patients. However, it is not clear whether sedation-induced states are similar to the biological sleep. We explored if sedative-induced states resemble biological sleep using multichannel electroencephalogram (EEG) recordings.

**Methods:**

Multichannel EEG datasets from two different sources were used in this study: (1) sedation dataset consisting of 102 healthy volunteers receiving propofol (N = 36), sevoflurane (N = 36), or dexmedetomidine (N = 30), and (2) publicly available sleep EEG dataset (N = 994). Forty-four quantitative time, frequency and entropy features were extracted from EEG recordings and were used to train the machine learning algorithms on sleep dataset to predict sleep stages in the sedation dataset. The predicted sleep states were then compared with the Modified Observer’s Assessment of Alertness/ Sedation (MOAA/S) scores.

**Results:**

The performance of the model was poor (AUC = 0.55–0.58) in differentiating sleep stages during propofol and sevoflurane sedation. In the case of dexmedetomidine, the AUC of the model increased in a sedation—dependent manner with NREM stages 2 and 3 highly correlating with deep sedation state reaching an AUC of 0.80.

**Conclusions:**

We addressed an important clinical question to identify biological sleep promoting sedatives using EEG signals. We demonstrate that propofol and sevoflurane do not promote EEG patterns resembling natural sleep while dexmedetomidine promotes states resembling NREM stages 2 and 3 sleep, based on current sleep staging standards.

## Introduction

Sedative drugs are often used to promote sleep [1–3]. However, the electroencephalographic (EEG) changes induced by first line sedatives include benzodiazepines and propofol differ from the EEG changes associated with natural sleep [4]. Benzodiazepines decrease sleep latency, SWS, and REM sleep stages [5]. Propofol spatially blurs EEG slow waves at low doses [6] and induces burst suppression in EEG at high doses [7, 8]. Dexmedetomidine causes EEG changes resembling natural sleep in a dose-dependent manner, suggesting utility in improving sleep quality in ICU patients [9–11]. However, these findings have been limited by the applied analyzing technology and the lack of direct comparison with EEG from natural sleep databases.

Polysomnography (PSG) is a gold standard method to evaluate sleep efficiency in patients and sleep technicians label the PSG into one of following five stages using the American Academy of Sleep Medicine (AASM) criteria: Wake, N1, N2, N3, and REM [12]. Each 30-second epoch is labelled into one of the five stages through visual analysis of the dynamic changes in EEG amplitude and frequencies based on standard AASM criterions defined using natural sleep recordings. This is a straightforward approach when sleep EEG recordings should be labelled in subjects not under the influence of any medication. Since sedatives alters the brain activity which results in change in the dynamics (time-frequency properties) of the EEG, the sleep technicians find it difficult to label sleep stages in these patients using standard AASM criteria and nearly 85% of the data cannot be labelled due to atypical EEG patterns [13]. This makes it difficult for the sleep technicians to determine the sleep/wake stages only based on the limited set of standard criterions. Due to this limitation, it is not possible to confirm whether the sedatives prescribed to a patient mimics/preserves biological sleep EEG patterns through visual analysis of the EEG.

As such, the aim of this study is to explore if states of sedation resemble biological sleep using multichannel EEG recordings or in other words “Which drugs are capable of promoting natural sleep?” using data-driven multidimensional analysis in order to overcome the limitations of previously applied technology. We developed a framework to identify biological sleep (based on the AASM criteria) promoting sedatives using EEG and machine learning algorithms. Since machine learning algorithms can be trained using multimodal features (instead of few hand-engineered amplitude and frequency features), we hypothesized that it is possible to identify sleep stages in sedated patients. We demonstrate the potential of this framework using propofol, sevoflurane and dexmedetomidine as prototype drugs using machine learning algorithms. It should be noted that in this study, the machine learning model was trained on the sleep data and later used to predict sleep stages on the sedation data. This should not be confused with the standard sleep staging or sedation level prediction problems where the model is trained, validated and tested within the same data types (for more details, please see the S1 File).

## Materials and methods

### Ethics statement

Prior approval was obtained from the Institutional Review Board of the University Medical Center Groningen to conduct the studies. Written informed consent was obtained from all the volunteers before participating in all study related activities. The clinical studies were registered at Clinical Trials.gov before including participants in the studies. Registration details are: NCT 02043938 (https://clinicaltrials.gov/ct2/show/NCT02043938, P.I.: MMRF Struys) (first inclusion: June 7^th^, 2014, study completion: June 4^th^, 2015) and NCT 03143972 (https://clinicaltrials.gov/ct2/show/NCT03143972, P.I. MMRF Struys) (first inclusion: September 1^st^, 2017, study completion: February 2^nd^, 2018).

### Sleep dataset

Publicly available multichannel Massachusetts General Hospital (MGH) sleep lab recordings [14] were used in this study. The MGH dataset (N = 994, mean age: 55± 14.4 years, M = 666, F = 328) consisted of 6-channel EEG recordings sampled at 200 Hz: Frontal (F3-M2 and F4-M1) central (C3-M2 and C4-M1) and occipital (O1-M2, O2-M1). Each 30s non-overlapping EEG segments were manually scored into five stages using standard American Academy of Sleep Medicine (AASM) guidelines: Wake (W), NREM stage 1(N1), stage 2 (N2), stage 3 (N3), and rapid eye movement (R).

### Sedation datasets

In total, 102 EEG recordings from 66 healthy volunteers were used in this study (UMCG dataset). Subject recruitment and data collection methods for propofol (N = 36, mean age: 41.9 ± 16.4 years, M = 16, F = 20), sevoflurane (N = 36 mean age: 41.9 ± 16.4 years, M = 16, F = 20) and dexmedetomidine (N = 30 mean age: 40.7 ± 15.8 years, M = 15, F = 15) have been described in our previous studies [15–18]. In healthy volunteers, EEG data for propofol and sevoflurane were recorded, initially using a 16 channel Neuroscan^®^ EEG monitor (Compumedics USA, Limited, Charlotte, NC, USA), with a sampling frequency of 5kHz, which was then lowered to 1kHz during file extraction and transition. For dexmedetomidine [18], a sampling rate of 5kHz was used to record a 17 channel EEG with a BrainAmp DC32 amplifier with a BrainVision recorder. We used six channels similar to MGH dataset for the analysis: Frontal (F3-M2 and F4-M1) central (C3-M2 and C4-M1) and occipital (O1-M2, O2-M1). Volunteers had their eyes closed for the entire study duration, across all groups. Weight less than 70% or more than 130% of ideal body weight, pregnancy, neurological disorders, diseases involving the cardiovascular, pulmonary, gastric, and endocrine system, recent use of psychoactive medication or intake of more than 20 g of alcohol daily were used as exclusion criteria. Sedation assessment was performed using the Modified Observer’s Assessment of Alertness/Sedation (MOAA/S) scale [19] by expert and trained anesthesiologists and any conflicts in the scoring were resolved through mutual assessment. MOAA/S score ranges from 5 to 0 where MOAA/S score = 5 corresponds to awake state (where the subject is in complete consciousness) and MOAA/S score = 0 corresponds to deep sedated state (where the subject is in completely unconscious). In between scores (4, 3, 2, 1) correspond to decreasing levels of consciousness (or increasing level of sedation).

Detailed description of the subject recruitment and data collection methods for propofol, sevoflurane and dexmedetomidine have been described in our previous publications [15, 16, 18]. In short, using a Fresenius Base Primea docking station (Fresenius-Kabi, Bad Homburg, Germany) controlled by RUGLOOPII software (Demed, Temse, Belgium) to steer target-controlled infusion (TCI), propofol was administered through an intravenous line. Effect-site concentration (CePROP) was predicted using the pharmacokinetic-dynamic (PKPD) model of Schnider et al [20]. A “staircase” step-up and step-down infusion method was used for propofol administration. The initial CePROP was set to 0.5 μg mL^-1^, followed by incremental steps toward target concentration of 1, 1.5, 2.5, 3.5, 4.5, 6 and 7.5 μg mL^-1^. Similar concentrations were targeted in the step-down phase. The proprietary algorithm of the Zeus® ventilator (Software version 4.03.35, Dräger Medical, Lübeck, Germany) was used to titrate and maintain a constant end-tidal sevoflurane concentration (ETSEVO). The initial ETSEVO was set to 0.2 vol%, which was gradually increased to 0.5, 1, 1.5, 2.5, 3.5, 4, 4.5 vol% with the upwards staircase method being followed by a downward staircase method using similar targeted concentrations. All steps were executed until tolerance or no motor response to all stimuli were noted, along with attainment of a significant burst suppression ratio of at least 40%. A 12 minutes equilibration time was maintained to achieve pharmacological steady state at each new step in drug titration once the desired effect-site concentration of propofol or the measured end-tidal vol% of sevoflurane was reached, followed by a 3-minutes measurement period.

A different protocol was utilized for dexmedetomidine. Effect-site TCI using the Hannivoort-Colin model [21–23] was used to administer Dexmedetomidine. Before administration, and once adequate function of all monitors, were confirmed, a 5 minute baseline measurement was done. Thereafter, dexmedetomidine infusion was initiated, with effect-site concentrations of 1.0 ng/ml (0–40 mins), 3.0 ng/ml (40–90 mins), 4.0 ng/ml (90–130 mins), 5.0 ng/ml (130–170 mins), and 6.0 ng/ml (170–200 mins). All effect sites reached steady state with the use of this protocol, and fifty minutes after increasing to a concentration of 6.0 ng/ml, dexmedetomidine infusion was stopped.

### Data preparation

**Fig 1:**
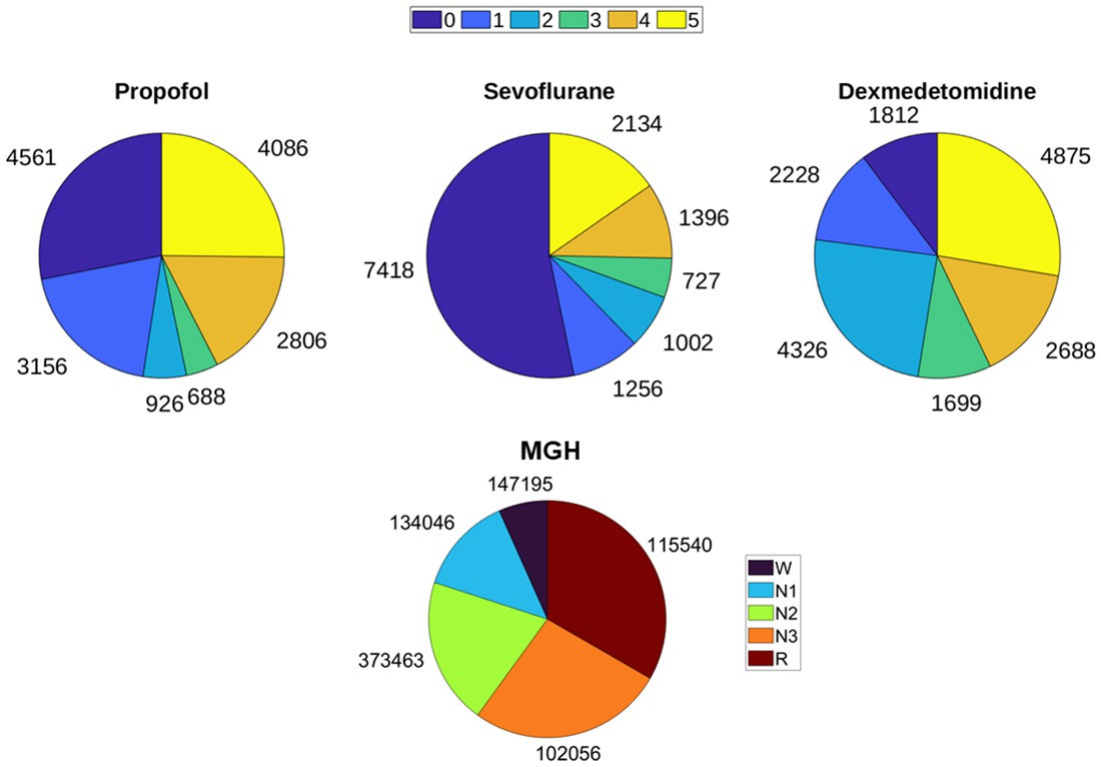
Distribution of 30s EEG epochs in sedation and sleep datasets used in this study. Similar number of MOAA/S epochs were present in all six channels in the sedation dataset.

Since EEG signals in all datasets were contaminated with artifacts, using a 30s non-overlapping window we excluded 30s EEG epochs satisfying any one of the following criterions from the analysis: (1) absolute amplitude of epochs >500 μV (movement artifacts); (2) epochs with 0 μV (flat EEG artifacts). EEG signals were then bandpass filtered between 0.1–25 Hz using a zero-phase 6^th^ order Butterworth bandpass filter and later resampled to 100 Hz to reduce computational complexity. We restricted the upper frequency to 25Hz to eliminate majority of muscle movement artifacts. EEG signals were then segmented into 30s epochs with the corresponding labels: five sleep stages in MGH and six MOAA/S scores in UMCG datasets. In case of UMCG dataset, 30s epochs after the time of MOAA/S scoring were used for the analysis since a steady-state sleep stage is only obtained after drug equilibration. The assigned MOAA/S score was designated for several minutes worth of subsequent EEG data until the next scoring was performed. The distribution of epochs in different groups in the sedation datasets is shown in Fig 1. Following number of epochs were obtained in the MGH dataset for each channel: W = 147195, N1 = 134046, N2 = 373463, N3 = 102056, and R = 115540. It should be noted that we included awake epochs from both datasets. Though wakefulness refers to the same state in both datasets, we believe it is important to include in both datasets because they were collected from two different devices and could have different resolution. In addition, for a given wake state, subjects could be in different levels of alertness and is necessary to normalize them for fair comparison.

### Feature extraction

From each 30S EEG epoch (in both sedation and sleep dataset), we extracted 44 quantitative EEG features from time, frequency and entropy domain summarized below:

*Time domain (12 features)*: Nonlinear energy operator, Activity (1^st^ Hjorth parameter), Mobility (2^nd^ Hjorth parameter), Complexity (3^rd^ Hjorth parameter) [24], Root mean square (RMS) amplitude, kurtosis, skewness; mean, standard deviation, skewness and kurtosis of amplitude modulation (AM) [25], Burst suppression ratio/minute (BSR) [26]; *Frequency domain (24 features)***:** mean power in delta (0.5–4 Hz), theta (4–8 Hz), alpha (8–12 Hz), spindle (12–16 Hz) and beta (16–25 Hz) bands; total spectral power (0.5–25 Hz), normalized band powers with respect to the total power, normalized band powers with respect to the delta band power, normalized band powers with respect to the theta band power; mean, standard deviation, skewness and kurtosis of frequency modulation (FM), spectral edge frequency, peak frequency; *Entropy domain (8 features)*: Singular value decomposition entropy [27], spectral entropy [28], state entropy [29], sample entropy [29], Renyi entropy [30], Shannon entropy [31], permutation entropy [32], and fractal dimension [33].

In this study, we utilized a set of 44 features that are commonly used in EEG based outcome prediction applications including automatic sleep-staging [34] and our previous work on a sedation level monitoring system [16, 17, 35]. Given their relevance and effectiveness in capturing dynamic changes of EEG signals related to sedation levels in time, frequency and entropy domains, we chose to incorporate the same set of features in this study. This decision was made to maintain consistency with our previous work and to leverage the established utility of these features in analysing EEG data for our current research objective.

### Machine learning framework

**Fig 2:**
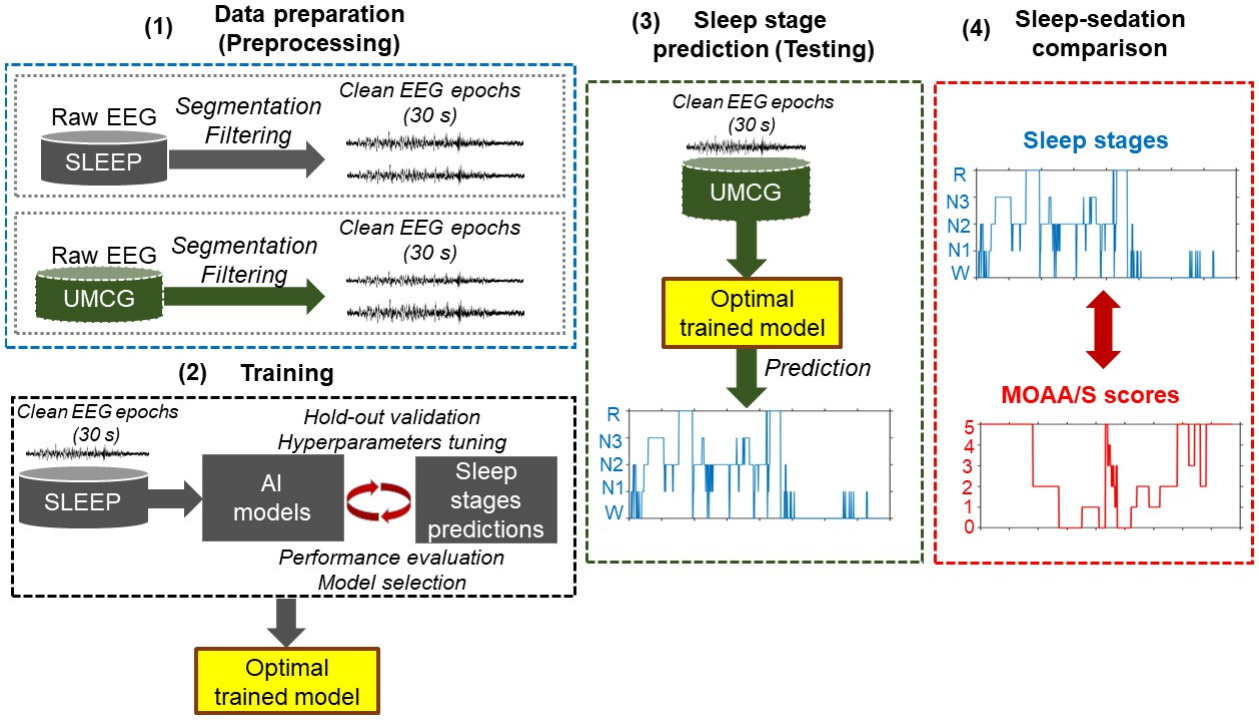
Illustration of the proposed artificial intelligence framework to predict sleep stages in sedation dataset. The raw EEG signal was first filtered and segmented into 30s epochs. After identifying the optimal model to classify different sleep stages on the MGH sleep dataset, it was later used to predict sleep stages in the UMCG sedation dataset. One-to-one comparison was then made between the MOAA/S scores and predicted sleep stages. This analysis was performed separately across all six channels.

The outline of the proposed framework is shown in Fig 2. The filtered EEG epochs from sleep dataset were used for training the ML models. The sleep dataset was further divided into 70% training set, 10% validation set and 20% test set. During training, features in the training set (MGH data) were normalized to have unit mean and standard deviation. Features in the validation (MGH data), test (MGH data) and sedation (UMCG) datasets were normalized w.r.t to the mean and standard deviation of the training set (MGH data). We performed grid-search to identify optimal model hyper-parameters and the model that provided highest prediction performance on the validation set was used to predict sleep stages in the test set. Here “hyperparameters” refer to the settings (or values) of the machine learning algorithms required to train the final model for the optimal and stable performance. Then, the final model was used to predict sleep stages in the UMCG dataset. Finally, the sleep stages were compared against the corresponding MOAA/S scores for each epoch.

We performed following experiments to evaluate the potential of the framework:

1. **Three class binary prediction**: In this approach, we grouped all NREM sleep stages (N1, N2, N3) into a single NREM sleep stage (N) and trained two binary classification models: WN = trained on W and N and WR = trained on W and R. Each model was then used to perform binary classification between awake and individual sedated states i.e WN was used to differentiate between MOAA/S = 5 and MOAA/S = 4 (M54); MOAA/S = 5 and MOAA/S = 3 (M53); MOAA/S = 5 and MOAA/S = 2 (M52); MOAA/S = 5 and MOAA/S = 1 (M51); MOAA/S = 5 and MOAA/S = 0 (M50). These steps were repeated using the WR model.
2. **Five class Binary prediction**: Like the previous step, the sleep stages were grouped into four sub-stages: WN1, WN2, WN3 and WR resulting in 4 machine learning models. Each model was then used to perform binary classification between awake and individual sedated states.

**Fig 3:**
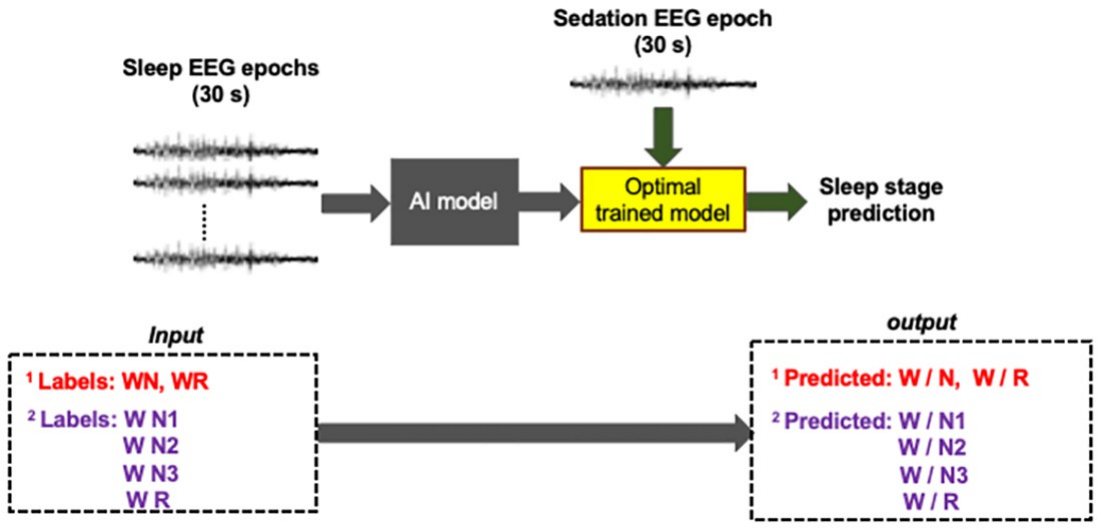
Illustration of different training and testing combinations for sleep stage predictions and correlation with sedation states developed in this study. The machine learning model was trained on sleep data to predict sleep stages on the sedation data which were then correlated with sedation states.

The above steps are illustrated in Fig 3. It should be noted that we did not group MOAA/S scores as the subject’s behavioral responses are very well defined and are significantly different within each score. Grouping could induce bias in the findings as our goal was to find specific sedated state correlating with individual sleep stages. Since in a typical sleep staging classification problem the sleep stages are predicted using different groupings, we employed similar strategy where three different classification schemes were performed to see the performance machine learning algorithms.

### Statistical analysis

We evaluated the potential of four traditional machine learning algorithms: elastic-net regularization based logistic regression [36], support vector machine, random forest and feed-forward neural networks in this study summarized in Table 1. Since the training set was highly imbalanced which can severely bias the performance of AI models, we used random under-sampling technique to balance the training set [37]. Predicted sleep stages for each 30s EEG epoch in the UMCG dataset were then compared with their corresponding MOAA/S score to identify relationship between predicted sleep stages and expert assessed sedation state.

**Table 1:** Model type, hyperparameters, grid search range and the final best performing parameters obtained during training the machine learning models to predict sleep stages.

Model type	Hyperparameters to tune	Grid search range (min,step size,max)	Best parameter
Elastic net logistic regression	α (Regularization)	0, 0.1, 1	0.8
Support vector machine with Gaussian kernel	γ = gaussian kernel, C = cost function	0.1, 0.1, 500	γ = 5.5, C = 100
Random forests	number of trees	50, 10, 1000	200
Neural networks	Number of layers/neurons	50,5, 1000	120

We first divided the sleep dataset into training, testing and validation subsets and estimated the performance of the individual classifiers for classification of sleep stages in the validation subset.

We used area under the receiver operator characteristic curve (AUC) as a metric to evaluate the performance of the machine learning models. We performed the analysis for all six channels separately and report the mean performance (and the interquartile range) across the six channels. All the coding and analysis were performed using MATLAB^®^ scripting language. Description of the algorithms and MATLAB functions are provided in the supplementary material.

## Results

### Identifying best performing ML model using sleep dataset

Table 2 shows the performance of the machine learning algorithms to differentiate between wake and other sleep stages during three and five class binary classification tasks on the test set. In both cases, the performance of the random forest algorithm outperformed the performances of other machine learning algorithms. All subsequent results will be based on the prediction obtained using the random forest model. The selected hyperparameters are shown in Table 1.

**Table 2:** The performance of the machine learning algorithms to differentiate between W and other sleep stages during three and five class binary classification tasks on the test set sleep stages during three and five class binary classification tasks on the test set.

	WN	WN1	WN2	WN3	WR
ENLR	0.75 (0.74–0.76)	0.64 (0.61–0.66)	0.79 (0.76–0.81)	0.82 (0.81–0.85)	0.80 (0.78–0.82)
SVMG	0.79 (0.77–0.80)	0.80 (0.78–0.83)	0.85 (0.83–0.87)	0.91 (0.90–0.93)	0.86 (0.83–0.97)
RFE	0.88 (0.86–0.91)	0.82 (0.81–0.84)	0.92 (0.91–0.94)	0.98 (0.96–0.99)	0.91 (0.90–0.93)
FNN	0.77 (0.75–0.78)	0.78 (0.77–0.80)	0.81 (0.80–0.83)	0.90 (0.88–0.94)	0.88 (0.85–0.90)

The random forest models outperformed other machine learning models in the classification task. The results are reported as mean (95% confidence interval) across six channels. **Abbreviations:** WN = trained on wake (W) and nonrapid eye movement (N) sleep stages; WR = trained on W and rapid eye movement R; WN1 = trained on W and N1; WN2 = trained on W and N2; WN3 = trained on W and N3; LR = Elastic-net based logistic regression; SVM = support vector machine with Gaussian kernel; RFE = Random forest; FNN = Feed-forward neural networks.

**Fig 4:**
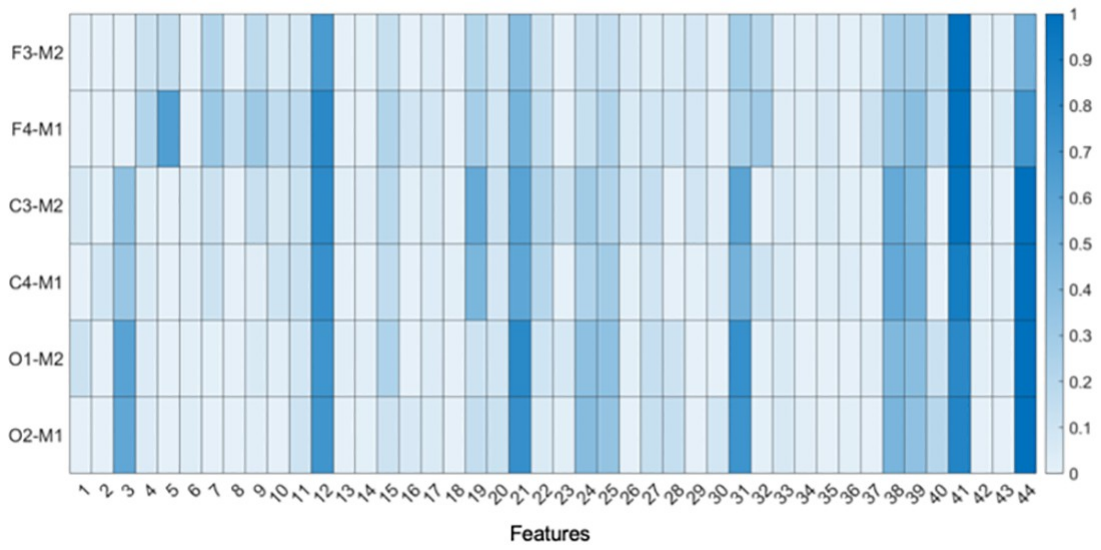
Heat map of the weights assigned to the 44 features by the random forest algorithm across all channels in the sleep data during training. The weights of the features with more discriminatory information are indicated with high intensity color (normalized to 1). Features 12, 21,31, 41 and 44 (burst suppression ratio, normalized alpha power with respect to the total power, normalized band powers with respect to the theta band power, Renyi entropy, and fractal dimension, respectively) were the top five discriminatory features.

Fig 4 shows the heatmap of the weights assigned to the random forest model across all channels. Five features from time, frequency and entropy domains were selected across all channels: BSR, normalized alpha power with respect to the total power, normalized band powers with respect to the theta band power, Renyi entropy, and fractal dimension.

### Binary prediction on the sedation dataset

**Fig 5:**
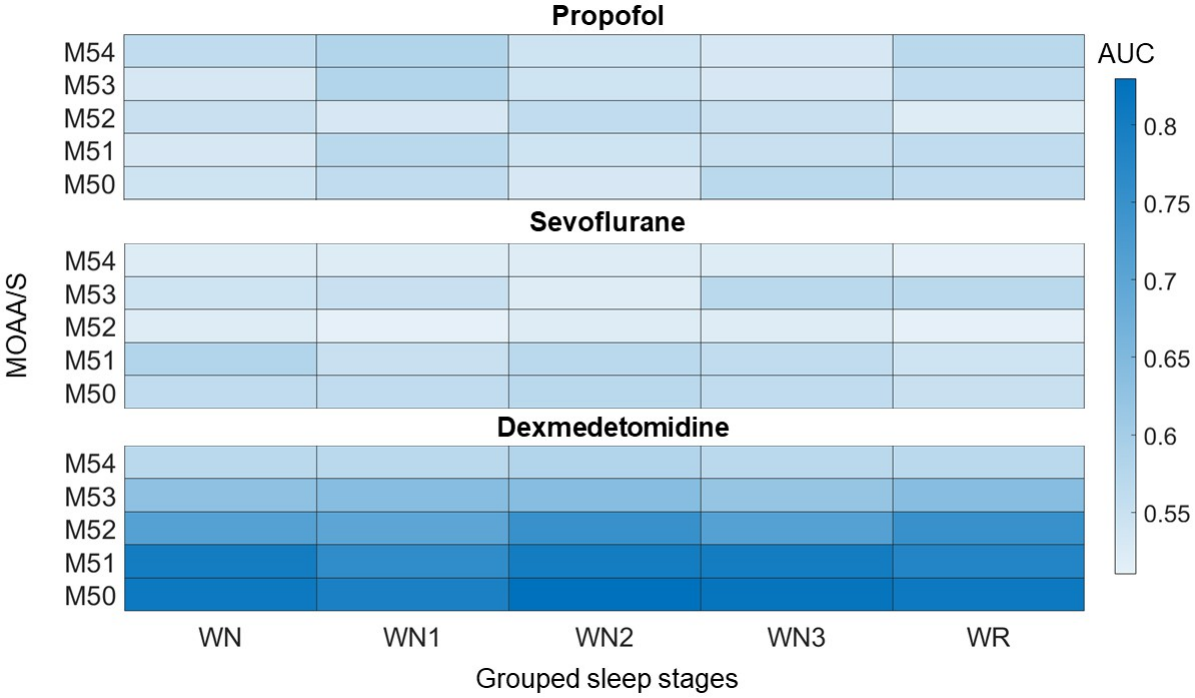
Heatmap of the performance of the random forest algorithm to differentiate between awake (MOAA/S score = 5) and different levels of sedation using sleep staging models for all three drugs. The colorbar represents the AUC values obtained by the model. Here WN = trained on wake (W) and nonrapid eye movement (N) sleep stages; WR = trained on W and rapid eye movement R; WN1 = trained on W and N1; WN2 = trained on W and N2; WN3 = trained on W and N3; MOAA/S = Modified Observer’s Assessment of Alertness/Sedation (MOAA/S) scale; M54 = MOAA/S 5 versus MOAA/S 4; M53 = MOAA/S 5 versus MOAA/S = 3; M52 = MOAA/S 5 versus MOAA/S = 2; M51 = MOAA/S 5 versus MOAA/S 1; M50 = MOAA/S = 5 versus MOAA/S = 0.

Fig 5 shows the heatmap of the prediction performance across different channels for all three drugs. The AUC values are provided in Table 3. In the case of three class classification, for both propofol and sevoflurane, the AUC’s of WN and WR models were less than 0.6. For dexmedetomidine, the WN and WR models provided highest performance to predict dexmedetomidine induced deep sedation (MOAA/S = 0) with an AUC’s between 0.74–0.78 across all channels. In the case of five class classification, the AUC’s of all models were less than 0.6 for propofol and sevoflurane. However, the AUC’s increased in a sedation-dependent manner for dexmedetomidine (WN3>WN2>WN1>WR) with the near similar performances of WN3 and WN2 models (AUC = 0.8). The F1- scores are provided in the supplementary material.

**Table 3:** The performance of the random forest algorithm to differentiate between awake (MOAA/S score = 5) and different levels of sedation using sleep staging models for all three drugs.

	DRUG	WN	WN1	WN2	WN3	WR
**M54**	***Propofol***	56.0 (51.07–59.97)	58.26 (56.99–59.54)	54.80 (50.45–59.55)	53.80 (50.52–57.46)	57.13 (56.30–57.96)
**M53**		53.53 (51.77–56.05)	58.52 (57.46–59.33)	54.58 (51.59–57.61)	53.66 (51.12–56.19)	56.92 (56.12–57.60)
**M52**		55.52 (54.19–56.45)	53.64 (52.99–54.54)	56.74 (55.84–57.86)	55.47 (50.85–58.02)	52.13 (50.18–53.41)
**M51**		53.27 (51.26–55.29)	57.11 (50.21–58.85)	54.12 (51.83–55.69)	55.21 (52.18–57.42)	56.80 (55.71–58.13)
**M50**		54.28 (52.21–56.63)	56.57 (50.51–59.56)	53.96 (52.37–55.96)	57.33 (55.66–59.86)	56.46 (55.34–57.46)
**M54**	***Sevoflurane***	52.68(51.73–53.86)	52.00(51.00–52.96)	52.35(51.80–52.78)	52.34(51.66–52.87)	51.91(51.21–52.26)
**M53**		54.41(50.01–59.90)	55.50(50.07–59.56)	52.53(50.6–56.84)	57.13(53.22–59.73)	57.17(50.07–59.82)
**M52**		52.54(52.11–53.12)	51.91(51.43–52.23)	52.51(52.04–52.91)	52.10(51.43–53.08)	51.37(50.99–52.00)
**M51**		58.22(57.47–59.07)	55.15(54.05–56.13)	57.58(56.68–58.56)	56.71(51.76–59.98)	54.39(52.87–54.98)
**M50**		56.30(51.25–58.55)	56.03(54.55–57.03)	57.22(55.48–59.63)	56.62(54.64–58.62)	55.26(54.25–56.38)
**M54**	***Dexmed***	57.56(57.13–58.12)	57.63(57.08–58.08)	58.38(57.86–59.09)	57.65(57.12–58.33)	57.26(56.95–57.82)
**M53**		63.38(62.48–64.38)	64.42(63.34–66.59)	64.53(62.93–65.84)	62.67(61.04–64.63)	64.02(62.95–65.60)
**M52**		71.58(66.01–77.07)	70.63(66.27–76.87)	75.53(69.51–78.08)	71.12(66.51–77.11)	75.14(69.97–77.29)
**M51**		80.43(79.40–81.36)	76.80(69.50–79.72)	80.77(79.30–81.87)	80.50(78.31–81.92)	78.91(77.78–80.14)
**M50**		81.96(79.82–83.30)	79.64(76.23–81.76)	83.22(81.47–84.66)	84.18(80.70–84.09)	73.56(70.65–82.91)

The results are reported as mean (95% confidence interval) across six channels.

**Abbreviations:** WN = trained on wake (W) and nonrapid eye movement (N) sleep stages; WR = trained on W and rapid eye movement R; WN1 = trained on W and N1; WN2 = trained on W and N2; WN3 = trained on W and N3; MOAA/S = Modified Observer’s Assessment of Alertness/Sedation (MOAA/S) scale; M54 = MOAA/S 5 versus MOAA/S 4; M53 = MOAA/S 5 versus MOAA/S = 3; M52 = MOAA/S 5 versus MOAA/S = 2; M51 = MOAA/S 5 versus MOAA/S 1; M50 = MOAA/S = 5 versus MOAA/S = 0.

## Discussion

This study quantitatively evaluated the biological sleep promoting characteristics of different sedatives based on multi-channel EEG using machine learning algorithms. There are three main findings in this study: (1) propofol- and sevoflurane-induced sedation does not mimic biological sleep EEG patterns, (2) to a certain extent dexmedetomidine induced sedation mimics biological NREM sleep EEG patterns in a sedation-dependent manner, and (3) special attention must be given to score sleep staging in patients under pharmacologically-induced sedation due to drug-induced changes in EEG dynamics as the EEG dynamics during sedated states (during propofol and sevoflurane infusion) did not correlate with any of the sleep stages.

### Sedatives and biological sleep

Propofol, sevoflurane and dexmedetomidine all evoke unconsciousness through modulation of different molecular targets that are located in a variety of neural networks in the brain and therefore, also evoke drug-dependent EEG features correlated to unconsciousness [38]. Propofol activates GABA_A_ receptors typically located in anatomical structures of the NREM-sleep-promoting pathways: respectively, the ventrolateral preoptic nucleus (VLPO) and the tuberomammillary nucleus [39]. Sevoflurane also activates GABA_A_ receptors, but in addition affects numerous other substrates that can primarily be linked to the cholinergic system, which is an important regulator of the sleep/wake cycle and promotes arousal during wakefulness and REM sleep [40]. The GABA_A_ activation of both propofol and sevoflurane evokes a generalized decrease of the firing rate of thalamocortical networks and consequently generates higher power (μV2) in the alpha (8-12Hz) and delta (0–4 Hz) wave frequency domains of the EEG spectrogram. Although some similarities in EEG features may be suspected when comparing drug-induced unconsciousness versus natural sleep (respectively the similar frequency of alpha wave activation versus sleep spindles), distinct differences in the spatiotemporal behavior between both conditions remain obvious [38].

In a study by Murphy et al. [6] slow waves in propofol were compared to slow waves recorded during natural sleep in eight subjects and it was observed that both populations of waves share similar cortical origins and preferentially propagate along the mesial components of the default network. However, it was also demonstrated that propofol slow waves were spatially blurred compared to sleep slow waves and failed to effectively entrain spindle activity. In our study, instead of visual analysis we used a data-driven approach capturing large heterogeneity seen in the sleep architecture. The machine learning algorithm did not find any similarity between sedation EEG and natural sleep EEG patterns during propofol and sevoflurane sedation at the population level.

In contrast, the mechanism of sedation with dexmedetomidine is mediated through a high affinity for G-protein coupled α_2_ adrenergic receptor, present in high density at the locus coeruleus (LC) [41]. The LC acts as a neuronal connected inhibitor of the sleep evoking VLPO, keeping VLPO inactive during wakeful conditions. The hypnotic effect of dexmedetomidine therefore results from an inhibition of the LC, and an indirect activation of the natural sleep promoting VLPO. Therefore, the resulting EEG features evoked by dexmedetomidine have more similarity to those evoked by natural sleep [42]. From Table 2, it is evident that EEG patterns during deep sedation state (M50) provides the highest AUC’s during WN2 and WN3 binary classification suggesting that the dexmedetomidine-induced deep sedation mimics NREM 2 and 3 sleep stages. Ideally a sedative should promote all stages of sleep for long-term healthy cognitive outcome. From the current analysis, though it is evident that dexmedetomidine promotes biological NREM sleep like EEG patterns, its effect on long-term sleep restorative properties is still unknown.

### Ambiguity in sleep scoring during sedation

Several previous studies have already analyzed the conventional polysomnography (PSG) recordings to identify sleep disruptions in the ICU patients under sedation and reduction in NREM stage 3 and REM sleep stages was observed [1]. This is due to the nature of the sedatives given to the patients to promote sleep. It was demonstrated that Propofol anesthesia is a sleep-like state and slow waves are associated with diminished consciousness even in the presence of high gamma activity [6]. On the contrary, in this study we showed that EEG patterns during propofol and sevoflurane do not mimic sleep EEG patterns at different dosage levels which is in line with the previous findings that sedative states are neurophysiologically distinct from sleep [43]. Due to this, sleep stage scoring in patients under sedation (such as ICU patients with multiple simultaneous multimodal drugs induction/infusion) is challenging due to drug induced atypical EEG patterns.

Similar to the previous findings, in this study we observed that the dexmedetomidine induced sedation mimics NREM sleep EEG patterns in a sedation-dependent manner(10,35). EEG patterns during dexmedetomidine sedation is characterized by slow oscillations (0–4 Hz) characterized by spindle-like activity as seen in NREM stage 3 sleep [44]. Our findings in this study also confirms that the dexmedetomidine induced deep sedation state promotes EEG patterns like NREM (stages 2 and 3) biological sleep. Propofol and sevoflurane sedation-induced states could confer benefits like sleep but have different EEG patterns compared to standard NREM and REM sleep EEG patterns.

### Future work

In this study, we aimed to address a critical clinical question: which anesthetic drugs produce EEG changes similar to those seen during physiologic sleep? To achieve this goal, we employed EEG data and utilized AI as a tool for analysis. Given the primary focus on answering this specific question, our evaluation primarily centered around assessing the performance of commonly used machine learning algorithms. This approach allowed us to determine the effectiveness of these algorithms in identifying the EEG patterns associated with sleep-like states induced by different anesthetic drugs. As this study serves as a proof-of-concept to demonstrate the feasibility of employing AI as a tool to solve a clinical problem, our focus was on establishing the basic efficacy of the AI framework rather than fine-tuning several model hyperparameters. The aim was to demonstrate the potential of AI in addressing a clinical problem. Therefore, we only conducted basic tuning of hyperparameters to demonstrate the framework’s ability to produce meaningful results.

In addition to undersampling technique, we also explored using both upsampling and synthetic minority oversampling (SMOTE) resampling techniques for our study. However, we observed that employing these methods would significantly increase the computational complexity of the algorithms. Given that our primary objective was to demonstrate the feasibility of utilizing AI to solve a clinical problem, the performance evaluation of various resampling techniques was beyond the scope of our proof-of-concept study.

The ability of deep learning models to model complex EEG patterns has indeed been a topic of ongoing research and discussion. Several recent studies demonstrate the effectiveness of deep learning in automated sleep stage scoring, highlighting the potential of these models in handling complex EEG patterns without the need for handcrafted features or extensive signal pre-processing [45–47]. While the results reported in the study are promising, it is essential to consider several factors when assessing the impact of using such models on final conclusions. Firstly, the generalizability of the model to different datasets and populations should be evaluated to ensure its robustness and reliability. Additionally, the interpretability of the model’s decisions and the ability to validate its findings against clinical standards are crucial for gaining trust and acceptance in clinical practice. Further research and validation studies are necessary to fully understand the implications and potential limitations of using deep learning models for EEG analysis.

## Limitations

There are several limitations of our study. First, we assumed that the EEG patterns during sedation states is relatively monolithic where the EEG dynamics of 30-second epochs are more uniform. Second, we used data from subjects undergoing sleep studies and the underlying comorbidities or medications could have altered the structure of EEG sleep patterns. Future work should involve training models using sleep data from the same individual undergoing sedation. Third, we tested the hypothesis on the volunteer dataset and the validation of the findings should be tested on the dataset from ICU cohorts. Fourth, we used machine learning algorithms in this study since the goal of this work was to explore if traditional machine learning algorithms could help us identify patterns using several time, frequency, and entropy features. Additional analysis using end-to-end deep learning algorithms that models complex EEG patterns could provide better insights on the correlation between sedation states and sleep stages. Future study should also look at the correlation of transition between individual sleep stages and sedation states instead of one-to-one correlation.

## Conclusions

We developed an EEG based data-driven framework to identify biological sleep promoting drugs using machine learning algorithms. We conclude that the sedation properties of propofol and sevoflurane do not promote biological sleep EEG patterns as defined by the AASM sleep scoring guidelines; dexmedetomidine promotes biological sleep EEG patterns in a sedation-dependent manner.

## Supporting information

S1 File (DOCX)
