# Supplementary material for: Do all sedatives promote biological sleep electroencephalogram patterns? A machine learning framework to identify biological sleep promoting sedatives using electroencephalogram
Source: PLoS One. 2024 Jul 2;19(7):e0304413. doi: 10.1371/journal.pone.0304413 (PMC11218986; doi:10.1371/journal.pone.0304413)
Supplement: S1 File — (DOCX) [file pone.0304413.s001.docx]

**Supplementary material 1.1:**

In this study, the machine learning model was trained on the sleep data and later used to predict sleep stages on the sedation data. Let **X** be the input signal and **Y** be the ground truth. In a typical sleep staging problem: **X**_1_= EEG, ECG or any physiological signal, **Y**_1_ = sleep staging annotations. The goal is to train an AI model on **X**_1_, **Y**_1_and predict unknown sleep stages **Ŷ**_1_ on the new or unseen **X̂**_1._ In a typical sedation level prediction problem: **X**_2_ = EEG, ECG or any physiological signal, **Y**_2_ = sedation states scores (MOAA/S etc.). The goal is to train an AI model on **X**_2_, **Y**_2_ and predict unknown sedation states **Ŷ**_2_ on the new or unseen **X̂**_2._

In our study we first trained AI model using **X**_1_ = Sleep EEG and **Y**_1_ = sleep stages to predict sleep stages (**Ŷ**_1_) on the SLEEP dataset. Next after obtaining the best model that predict sleep stages on the SLEEP dataset, we predicted sleep stages (**Ŷ**_1_) on the SEDATION dataset. Now we have two measurements vectors: one vector is the vector of sedation scores assigned by an anesthesiologist (**Y**_2_). The second vector is the vector of sleep stages predicted by the AI algorithm (**Ŷ**_1_). We then performed a “one-to-one comparison between the predicted sleep stages on the sedation dataset and sedation levels scored by expert anesthesiologists.”

**Supplementary material 1.2:** List of functions used in MATLAB for training machine learning algorithms:

| **Algorithm** | **MATLAB inbuilt function** |
| --- | --- |
| Elastic net regularization | lasso |
| Support vector machine | fitcsvm |
| Random forest | fitcensemble |
| Feed-forward neural networks | feedforwardnet |

**Supplementary material 1.3**: The following strategy was used to train machine learning models on sleep data:

| **Model** | **Strategy** |
| --- | --- |
| WN1 | Train the model on W and N1 epochs from sleep data. Predict sleep labels on sedation data. |
| WN2 | Train the model on W and N2 epochs from sleep data. Predict sleep labels on sedation data. |
| WN3 | Train the model on W and N3 epochs from sleep data. Predict sleep labels on sedation data. |
| WR | Train the model on W and R epochs from sleep data. Predict sleep labels on sedation data. |
| WN | Train the model on W and N (N1+N2+N3) epochs from sleep data. Predict sleep labels on sedation data. |

**Supplementary figure 1:** The performance (% F-scores) of the Random forest algorithm to differentiate between awake (MOAA/S score = 5) and different levels of sedation using sleep staging models for (a) Propofol,(b) Sevoflurane, and (c) Dexmedetomidine. The results are reported as mean across six channels

**Abbreviations:** WN = trained on wake (W) and nonrapid eye movement (N) sleep stages; WR = trained on W and rapid eye movement R; WN1 = trained on W and N1; WN2 = trained on W and N2; WN3 = trained on W and N3; MOAA/S = Modified Observer’s Assessment of Alertness/Sedation (MOAA/S) scale; M54 = MOAA/S 5 versus MOAA/S 4 ; M53 = MOAA/S 5 versus MOAA/S = 3; M52 = MOAA/S 5 versus MOAA/S = 2; M51 = MOAA/S 5 versus MOAA/S 1 ; M50 = MOAA/S = 5 versus MOAA/S = 0.

**
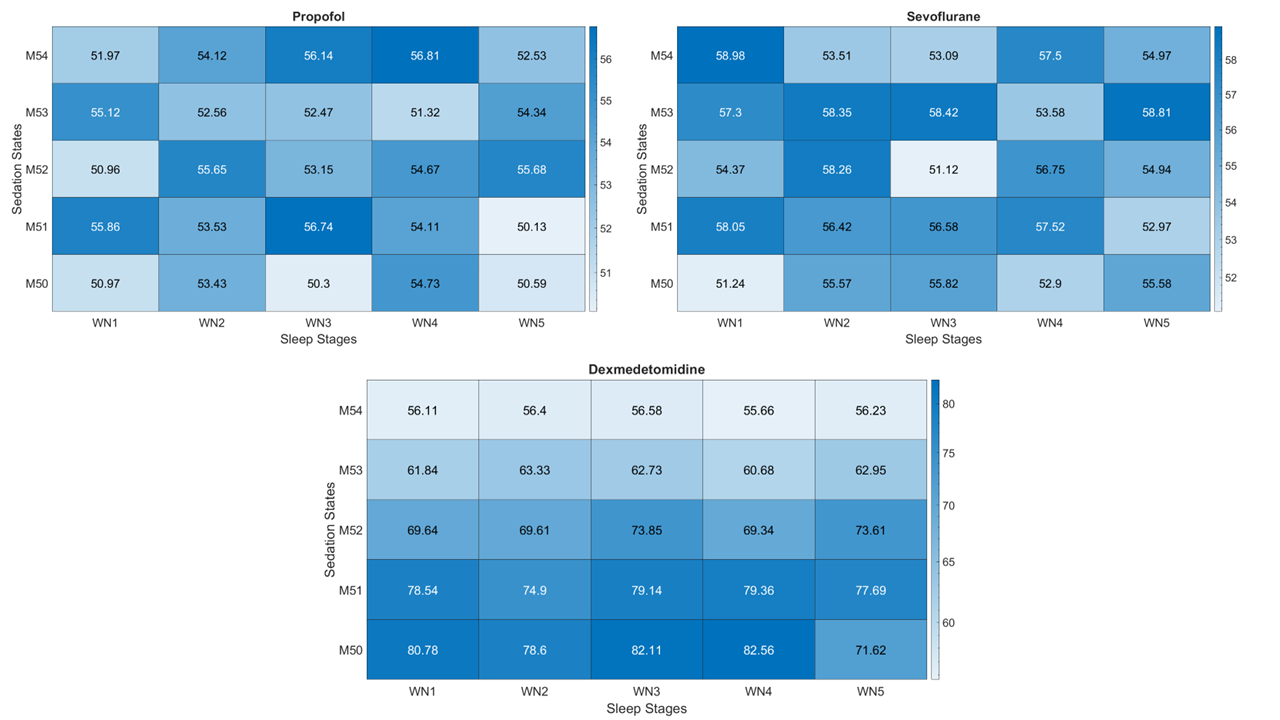
**
